# Targeted Delivery of GP5 Antigen of PRRSV to M Cells Enhances the Antigen-Specific Systemic and Mucosal Immune Responses

**DOI:** 10.3389/fcimb.2018.00007

**Published:** 2018-01-25

**Authors:** Luping Du, Zhengyu Yu, Fengjiao Pang, Xiangwei Xu, Aihua Mao, Wanzhe Yuan, Kongwang He, Bin Li

**Affiliations:** ^1^Key Laboratory of Veterinary Biological Engineering and Technology Ministry of Agriculture, Institute of Veterinary Medicine, Jiangsu Academy of Agricultural Sciences, Nanjing, China; ^2^Jiangsu Co-infection Center for Prevention and Control of Important Animal Infectious Disease and Zoonoses, Yangzhou, China; ^3^Institute of Animal Immunity Engineering, Jiangsu Academy of Agricultural Sciences, Nanjing, China; ^4^College of Animal Medicine, Agricultural University of Hebei, Baoding, China

**Keywords:** PRRSV, PLGA, M cells, DNA vaccine, immune response, delivery system

## Abstract

Efficient delivery of antigens through oral immunization is a first and critical step for successful induction of mucosal immunity, which can provide protection against pathogens invading the mucosa. Membranous/microfold cells (M cells) within the mucosa can transcytose internalized antigen without degradation and thus play an important role in initiating antigen-specific mucosal immune responses through inducing secretory IgA production. In this research, we modified poly (D, L-lactide-co-glycolide) (PLGA) nanoparticles (NPs) with *Ulex europaeus* agglutinin 1 (UEA-1) and successfully prepared an oral vaccine delivery system, UEA-1/PLGA NPs. PLGA NPs were prepared using a standard double emulsion solvent evaporation technique, which can protect the entrapped PRRSV DNA vaccine [pcDNA3.1-SynORF5 (synthetic ORF5)] or subunit vaccine ORF5-encoded glycoprotein (GP5) from exposure to the gastrointestinal (GI) tract and release the plasmids in a controlled manner. With UEA-1 modification, the UEA-1/PLGA NPs can be effectively transported by M-cells. We investigated immune response induced by UEA-1/PLGA-SynORF5 or UEA-1/PLGA-GP5 following inoculation in mice and piglets. Compared with PLGA-SynORF5 or PLGA-GP5 NPs, UEA-1/PLGA-SynORF5, or UEA-1/PLGA-GP5 NPs stimulated significantly increased serum IgG levels and augmented intestinal IgA levels in mice and piglets (*P* < 0.05). Our findings indicate UEA-1/PLGA NPs can be applied as a promising and universally robust oral vaccine delivery system.

## Introduction

Porcine reproductive and respiratory syndrome (PRRS) is one of the viral diseases causing devastating economic losses to the swine industry worldwide (Du et al., 2013). The causative agent, porcine reproductive and respiratory syndrome virus (PRRSV), infects pigs mainly through mucosal surfaces. Therefore, induction of mucosal immunity as well as systemic immunity is essential to inhibit PRRSV entry.

At present, commercial PRRSV vaccines including killed and modified live vaccines are available. However, these vaccines have inherent disadvantages. Killed vaccines are weakly immunogenic and modified live vaccines have the potential to revert to high virulence. Importantly, commercial PRRSV vaccines are administrated through intramuscular immunization (i.m.), which fails to induce efficient mucosal immune responses to prevent PRRSV entry through mucosal surfaces. Although nasal or oral mucosal immunization could induce mucosal immune responses, nasal immunization is more difficult than oral vaccination in pigs in large-scale immunizations. Moreover, the application of nanotechnology in the development of particle-mediated delivery systems for vaccines solves the challenges of oral vaccinations, such as the low pH environments and enzymatic degradation (Eldridge et al., 1989; Gupta et al., 1998; Singh et al., 2000). Biodegradable and biocompatible poly (D, L-lactide-co-glycolide) (PLGA) nanoparticles (NPs) are non-toxic, safe to use and have been approved by the United States Food and Drug Administration (Du et al., 2015). Nps prepared from PLGA containing hepatitis B virus, rotavirus, influenza virus, and parainfluenza virus delivered to mucosal sites in mice have been shown to generate a protective immune response (Thomas et al., 2011). Additionally, a PLGA-entrapped PRRSV vaccine has been demonstrated to significantly increase immune responses and protection (Dwivedi et al., 2013; Binjawadagi et al., 2014a,b).

The discovery of membranous/microfold cells (M cells), located in the follicle-associated epithelium (FAE) overlying Peyer's patches (PP), has made it possible to induce efficient mucosal immunity. Antigen transcytosis by M cells transports the antigen from the gut lumen to underlying lymphoid tissues, thereby generating a mucosal immune response (Neutra et al., 1996). *Ulex europaceous* agglutinin 1 (UEA-1), specific for α-L-fucose residues, can selectively bind to M cells (Kessimian et al., 1986). To increase the transport efficiency of NPs across the intestinal barrier to the PP, we used UEA-1 to modify NPs.

In this study, we successfully developed a PRRSV DNA vaccine entrapped in PLGA NPs modified with UEA-1 (UEA-1/PLGA-SynORF5). Enhanced mucosal and systemic immune responses were observed following inoculation of mice with the construct UEA-1/PLGA-SynORF5. Even though UEA-PLGA-GP5 also induced improved mucosal and systemic immune response than PLGA-GP5 in mice, significant higher levels of systemic IgG and mucosal IgA antibody were observed in the group receiving UEA-1/PLGA-SynORF5, so we chose UEA-1/PLGA-SynORF5 to evaluate the immune response following inoculation in piglets. And as expected, improved mucosal and systemic immune responses were observed following inoculation of piglets with the construct UEA-1/PLGA-SynORF5. Our findings suggest PLGA NPs immobilized with UEA-1 may be an effective carrier for the oral vaccination.

## Materials and methods

### Materials

Poly (D,L-lactide-co-glycolide) (PLGA, acid terminated, lactide: glycolide 75: 25, Mw 4,000–15,000), Poly (vinyl alcohol) (PVA) (Mw 9,000–10,000, 80% hydrolyzed), N-(3-dimethylaminopropyl)-N-ethylcarbodiimide hydrochloride (EDC), 2-(N-morpholino) ethanesulfonic acid, 4-morpholineethanesulfonic acid monohydrate (MES), coumarin-6 and lectin from *Ulex europaeus* (UEA-1) were purchased from Sigma–Aldrich (St. Louis, USA). 4, 6-diamidino-2-phenylindole (DAPI) was obtained from Invitrogen (CA, USA).

### Plasmids and proteins

Plasmid pcDNA3.1-SynORF5, maintained in our laboratory, based on the native ORF5 gene of HP-PRRSV strain JSKM (GenBank accession number HQ832104) was constructed as previously described (Li et al., 2009). HP-PRRSV strain JSKM, isolated from the lungs of a pig infected with the “high fever” syndrome in Jiangsu Province, was propagated and titrated in Marc-145 cells as previously described (Lewis et al., 2010). Large-scale preparations of plasmid pcDNA3.1-SynORF5 were purified by Endofree Maxi Plasmid Kit (TIANGEN Biotech, Beijing, China) as per the manufacturer's instructions. Plasmids were adjusted to a final concentration of 5 μg/μL.

PRRSV GP5 protein was prepared and maintained in our laboratory as previously described (Fang et al., 2006). Proteins were adjusted to a final concentration of 2 μg/μL.

### Preparation of PLGA-SynORF5 and PLGA-GP5 NPs

PLGA-SynORF5 and PLGA-GP5 NPs were prepared using a modified double-emulsion solvent evaporation method as previously described (Cao and Shoichet, 1999; Capan et al., 1999; Soderquist et al., 2010). First, 300 mg PLGA (75:25) were dissolved in 2 mL dichloromethane, which was used as the O phase; 500 μL plasmid pcDNA3.1-SynORF5 (5 μg/μL) or 500 μL protein GP5 were dissolved in 500 μL PVA (concertration 5% (w/v)), which was used as the W1 phase. The W1 phase was added to the O phase and an emulsion was formed by homogenizing at 15,000 rpm for 20 s using a T18 homogenizer (IKA, German) in an ice bath. Second, the emulsion was poured into 50 mL 5% PVA solution and homogenized for 30 s at 12,000 rpm. Subsequently, the preparation was stirred overnight at room temperature (RT) to remove the organic solvent. Finally, NPs were washed in distilled water three times by centrifugation at 10,000 rpm for 30 min.

### Preparation of coumarin-6-loaded PLGA NPs (PLGA-coumarin-6 NPs)

PLGA-coumarin-6 NPs were prepared as described previously (Jiang et al., 2014). Briefly, a sodium oleate solution prepared in distilled water (W1 phase) was emulsified with PLGA along with of coumarin-6 dissolved in 2 mL of methylene chloride (O phase) to form a stable initial emulsion (W1/O). Further processes were performed similar to the preparation of the PLGA-GP5 NPs as described above.

### Preparation of UEA-1 modified PLGA-SynORF5, -GP5 or coumarin-6 NPs

UEA-1/PLGA-SynORF5, UEA-1/PLGA-GP5, or UEA-1/PLGA-coumarin-6 NPs were prepared via modified carbodiimide chemistry (Keegan et al., 2006; Li et al., 2011). Briefly, PLGA-SynORF5, PLGA-GP5 or PLGA-coumarin-6 NPs were suspended in 0.5 mL 0.1 M MES buffer (pH 5.5–6.7), then, carboxylate-groups were activated by EDC dissolved in 0.5 mL MES buffer. After end-to-end incubation for 15 min at room temperature, the NPs were washed three times with MES buffer to remove any unreacted EDC and resuspended in 1 mL 0.2 M borate buffer (pH 8.5). UEA-1 (500 μg) was then added followed by gentle end-to-end mixing for 4 h. Resultant UEA-1/PLGA-SynORF5, UEA-1/PLGA-GP5 or UEA-1/PLGA-coumarin-6 NPs were centrifuged for 10 min at 12,000 rpm and the supernatant was collected for determination of the amount of unbound UEA-1.

### Characterization of PLGA-SynORF5 and PLGA-GP5 NPs

#### Determination of size and morphology of NPs

The particle size and morphology of NPs were determined by scanning electron microscopy (SEM) (Shau et al., 2012). Briefly, a drop of samples was placed onto a copper grid mounted on an aluminum stage. After air-drying, samples were coated with gold/palladium under vacuum (20 mA, 120 s) using an ion sputter coater EMS150R S (Electron Microscopy Sciences, USA). SEM was performed with a JSM-5610 LV scanning electron microscope (JEOL, Japan) at an accelerating voltage of 10 kV.

#### Evaluation of entrapment efficiency (EE) of pcDNA3.1-SynORF5 and GP5

The amount of pcDNA3.1-SynORF5 plasmids loaded into PLGA NPs was determined as follows (Zhao et al., 2010). Lyophilized PLGA-SynORF5 NPs were immersed in 1 mL dichloromethane, to which 5 mL 0.1 M PBS (pH 7.4) were added. The mixture was stirred for 30 min followed by centrifugation at 4,000 rpm for 8 min. The supernatant was collected and the concentration of pcDNA3.1-SynORF5 was determined by measuring absorbance at 260 nm using Biomate 3S UV-Visible Spectrophotometer (Thermo Scientific, USA).

The amount of entrapped GP5 protein in NPs was determined as described previously (Corrigan and Li, 2009). Briefly, freeze-dried NPs were dispersed into 3 mL 0.1 M NaOH containing 5% (w/v) SDS. The suspension was incubated in a water bath at 60°C for 1 h. Following centrifugation at 10,000 rpm for 10 min, the concentration of the GP5 protein in the supernatant was measured by using RC DC^TM^ Protein Assay (Bio-Rad, USA) for micro-BCA analysis.

The EE of the NPs were calculated using this formula (Park et al., 2003; Manca et al., 2008):

$$\text{EE}(\%)\;=\;({\text{m/m}}_{0})\times \text{100}\%\;=\;{\text{CV/m}}_{0}$$

Where m is the mass of the pcDNA3.1-SynORF5 or GP5 loaded in PLGA NPs and C and V are the concentration and volume of the supernatant, respectively. m_0_ is the initial amount of pcDNA3.1-SynORF5 or GP5.

### *ex vivo* ligated ileal loop assay

The *ex vivo* ligated ileal loop assay was performed as previously described (Primard et al., 2010; Ma et al., 2014) with some modifications. Briefly, female BALB/c mice (6–8 weeks old) were fasted overnight and anesthetized by intraperitoneal injection of pentobarbital sodium (50 mg/kg animal weight). Subsequently, 2 cm of the ileal loop containing a PP was ligated and injected with PLGA-coumarin-6 NPs or UEA-1/PLGA-coumarin-6 NPs diluted in PBS (10 mg/mL). After 1-h incubation, mice were euthanized by cervical dislocation. The ligated ileal loop was excised and washed with PBS, followed by fixation with 4% paraformaldehyde for 1 h at room temperature. Frozen sections (10 μm) of PP were obtained using a CM1950 cryostat (Leica Microsystems, Wetzlar, Germany). Tissue samples captured on Superfrost plus microscope slides (Thermo Scientific, USA) were washed with PBS three times to remove any residual optimal cutting temperature compound. Samples were then blocked with PBS containing 5% FBS and stained with DAPI. CLSM images were recorded using an Ultra View VOX CLSM instrument (PerkinElmer, USA).

### Animal study design

#### Experiment in mice

Female BALB/c mice (6–8 weeks old, *n* = 49 total) were purchase from Yang Zhou University. Mice were randomly divided into seven groups (*n* = 7 mice per group) and acclimated under controlled specific-pathogen-free conditions. The vaccination protocol in this study was as follows. Each group was immunized twice at 2-week intervals. Three groups were given an oral gavage of 100 μL PBS containing an amount equivalent to 100 μg pcDNA3.1-SynORF5 in pcDNA3.1-SynORF5 solution, PLGA-SynORF5 or UEA-1/PLGA-SynORF5. Three groups were given an oral gavage of 100 μL PBS containing an amount equivalent to 100 μg of GP5 in GP5 solution, PLGA-GP5 or UEA-1/PLGA-GP5. One control group was orally immunized with 100 μL PBS following the same immunization protocol. Sera were collected at 14 and 28 dpi for serological tests. At 42 dpi, mice were euthanized and samples of intestine (duodenum to rectum, 3 cm) were excised and ground using a Fast Prep-24 instrument (MP Biomedicals, USA) in 500 μL 0.01 M PBS. The obtained slurry was then centrifuged at 1,200 rpm for 30 min at 4°C and the supernatant was collected for IgA titer estimation (Shimosato et al., 2011).

#### Experiment in pigs

Twenty piglets weaned at 3 weeks of age were obtained from a PRRS-free farm in Nanjing. Piglets were confirmed to be negative for PRRSV by PCR and ELISA (IDEXX, USA). Piglets were then randomly separated into four groups and housed in separate rooms at the animal facility of the Institute of Veterinary Medicine, Jiangsu Academy of Agricultural Sciences, Nanjing, Jiangsu Province. Three groups were orally vaccinated twice at 2-week intervals with pcDNA3.1-SynORF5 PLGA-SynORF5 or UEA-1/PLGA-SynORF5 dissolved in 1 mL PBS, each containing 500 μg plsamids. The control group was orally immunized with 1 mL PBS. Sera were collected from each piglet at 14, 28, and 42 dpi to detect specific anti-PRRSV antibodies. Fecal samples were collected at 14, 28, and 42 dpi. Approximately 0.5 g feces were diluted in PBS buffer, followed by vortex and centrifugation at 1,200 rpm for 30 min at 4°C. The supernatant was collected for IgA titer estimation. At 42 dpi, all piglets were euthanized for intestinal lavage fluid IgA antibody detection.

### Serological tests

Serum, intestinal lavage fluid and fecal samples obtained from mice and piglets were analyzed for PRRSV-specific IgG or IgA antibody responses by enzyme-linked immunosorbent assay (ELISA) (Li et al., 2009). Briefly, 96-well microplates were coated with 100 μL GP5 suspended in 0.05 M carbonate sodium buffer (pH 9.6). After incubation overnight at 4°C, microplates were washed three times with 0.05% Tween 20 in PBS, and then blocked with 100 μL 5% skimmed milk powder in PBST for 2 h at 37°C. After three times washes, 2-fold serial dilutions of serum (starting with 1:80) or intestinal wash samples or fecal samples (start with 1:20) were added to the microplates, followed by incubation for 90 min at 37°C and washed three times. Subsequently, 100 μL horseradish peroxidase (HRP)-conjugated goat anti-mouse or anti-pig IgG (BETHYL, USA) or HRP-conjugated goat anti-mouse or anti-pig IgA (BETHYL, USA) were diluted to 1:10,000 in PBST containing 2.5% skimmed milk, seeded in each well and incubated for 45 min at 37°C. After another three washes with PBST, wells were incubated with 3, 3', 5, 5″-Tetramethylbenzidine (TMB) substrate solution (Biopanda Diagnostics, UK) at 37°C for 15 min, and the reaction was stopped by adding 2 M H_2_SO_4_. The OD_450_ was determined by an ELISA reader (BioTek, USA). PRRSV-specific IgG or IgA antibody titers were expressed as the reciprocal of the highest dilution of serum or intestinal lavage fluid producing ratio values of 2.1.

Serum neutralization assays were performed as described by Ostrowski et al. (2002). Briefly, the collected sera samples were heat-inactived for 30 min at 56°C, and serially diluted in two fold. Then the diluted samples were mixed with equal volume of HP-PRRSV strain JSKM containing 100 TCID_50_ and incubated at 37°C for 1 h. The mixtures were added to Marc-145 monolayers in 96-well tissue culture plates, and incubated at 37°C with 5% CO_2_, cells were examined daily up to 5 days for cytopathic effects (CPE). The neutralization titers were expressed as the reciprocal of the highest serum dilution resulting in complete neutralization. Each sample was run in triplicate.

### Statistical analysis

Statistical analyses were performed using GraphPad Prism version 5 (GraphPad Software, San Diego, CA, USA). Comparisons between groups were performed using student's *t*-tests and one-way analysis of variance. A value of *P* < 0.05 represents a statistically significant difference. All data are expressed as the mean ± standard error of mean (s.e.m.).

### Ethics approval

The study and protocol was approved by the Science and Technology Agency of Jiangsu Province. All animal experiments were performed with the approval of the Jiangsu Academy of Agricultural Sciences Experimental Animal Ethics Committee (NKYVET 2015-0066) in accordance with relevant guidelines and regulations. All efforts were made to minimize animal suffering.

## Results

### Characterization of pcDNA3.1-SynORF5 or GP5 loaded PLGA NPs

**Figure 1:**
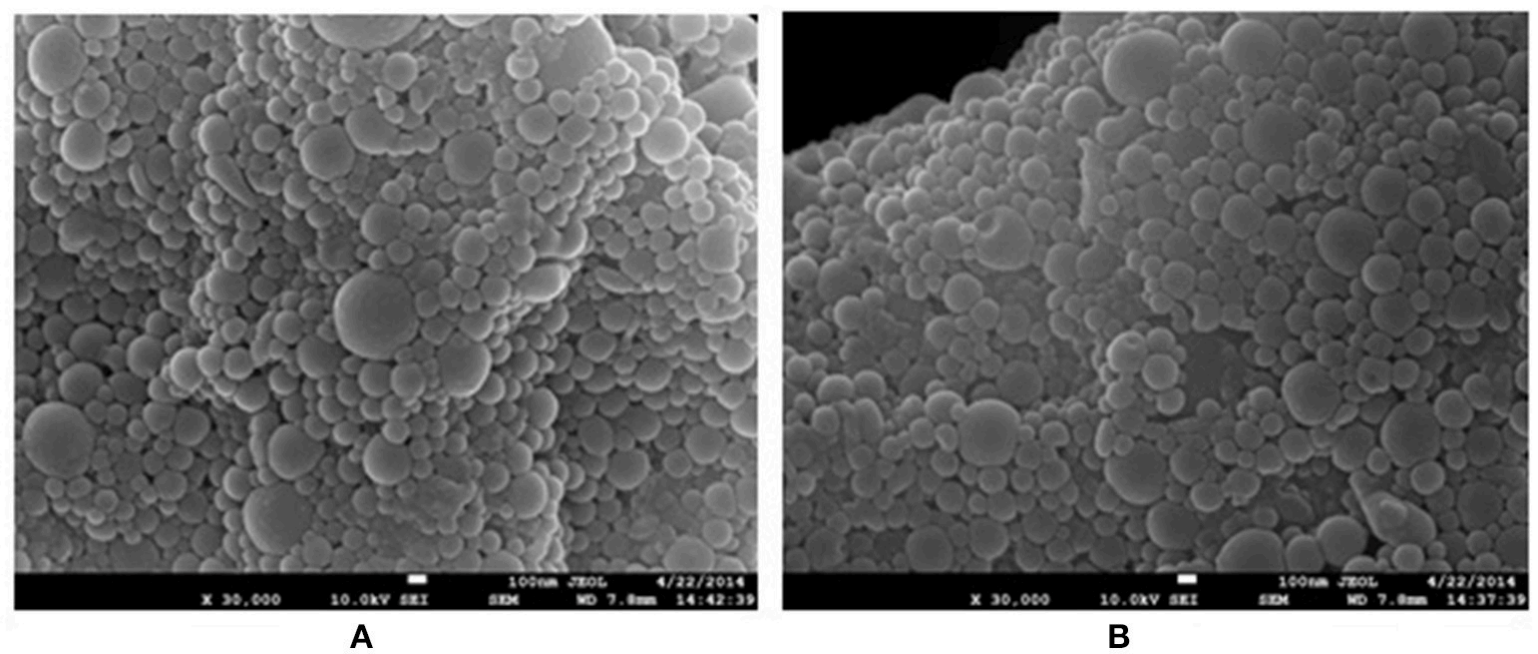
SEM image of PLGA-SynORF5 NPs **(A)** and PLGA-GP5 NPs **(B)**, showing their size and morphology.

NPs used in this study were characterized for size and morphology. The sizes of PLGA-SynORF5 and PLGA-GP5 NPs ranged 100–600 nm (Figure 1). The proportion of PLGA NPs with large size (range 500–600 nm) was much lower than the proportion of PLGA NPs with small size (range 100–200 nm). NP surface morphology was spherical with no surface discontinuity (Figure 1). The EE of pcDNA3.1-SynORF5 or GP5 in PLGA NPs were equal to (52.24 ± 1.32%) and (97.47 ± 1.22%), respectively. The ratio of conjugated UEA-1 to the total mass of UEA-1 was 67.81 ± 1.83%.

### *ex vivo* PP transportation

**Figure 2:**
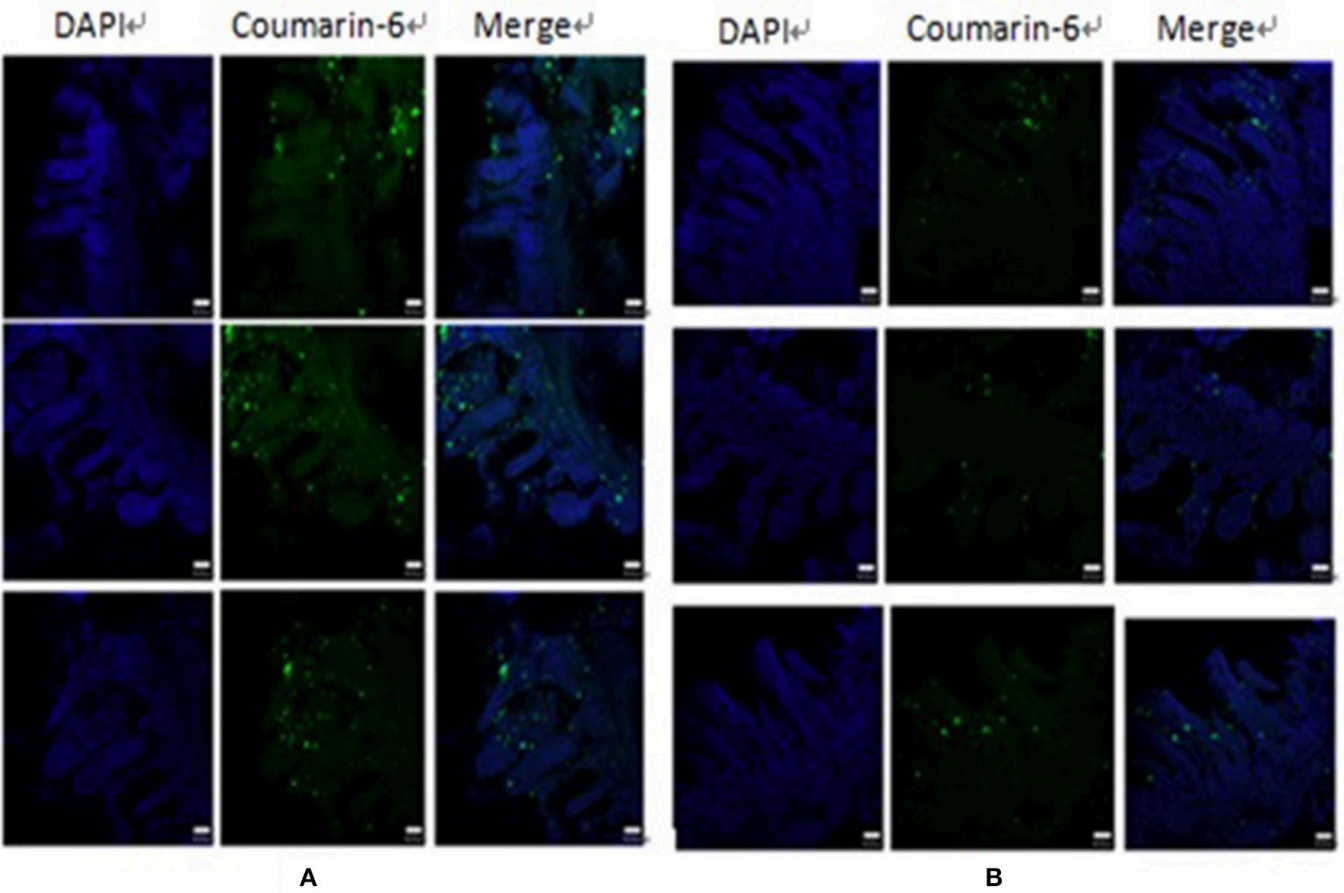
CLSM images of the effect of UEA-1 conjugation on M cell transport. UEA-1/PLGA-coumarin-6 NPs **(A)** and PLGA-coumarin-6 NPs **(B)** penetrated the intestine mainly in the PP.

*Ex vivo* ligated ileal loop assays were performed to investigate whether UEA-1 modified PLGA NPs with the ability to target M cells can increase antigen uptake efficiency. UEA-1/PLGA-coumarin-6 or PLGA-coumarin-6 NPs were incubated *ex vivo* in the ligated ileal loop for 1 h. PP cryosections were then prepared for confocal laser scanning microscopy (CLSM) observations. As shown in Figure 2, blue and green fluorescent signals represent the cell nucleus and coumarin-6 loaded PLGA NPs, respectively. There were more green signals captured in the sample treated with UEA-1 modified PLGA-coumarin-6 NPs compared with the sample treated with PLGA-coumarin-6 NPs, suggesting that UEA-1/PLGA NPs penetrated more efficiently into the PP than PLGA NPs without any ligands. Therefore, UEA-1 modified PLGA-coumarin-6 NPs could efficiently transport antigen across the intestinal barrier to the PP.

### Systemic and mucosal immune response in mice

**Figure 3:**
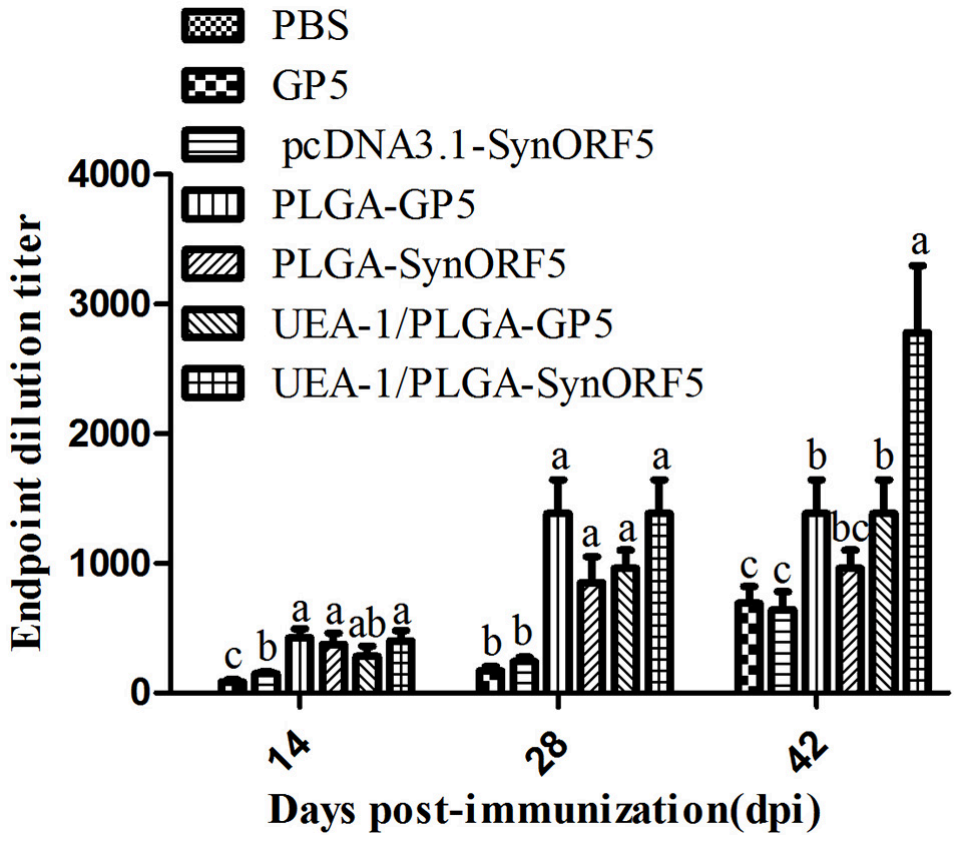
Systemic immune response in mice induced by different PRRSV vaccine formulations. Female BALB/c mice (6–8 weeks old, *n* = 7 in each group) were orally immunized with 100 μL of PBS, pcDNA3.1-SynORF5, GP5, PLGA-SynORF5, PLGA-GP5, UEA-1/PLGA-SynORF5, or UEA-1/PLGA-GP at 0 and 2 weeks. Sera were collected at 14, 28, and 42 dpi to determine GP5-specific IgG antibody by ELISA. Data represent the mean and s.e.m. of 7 mice per group. Columns with different letters differ significantly (*P* < 0.05).

Sera collected at 14, 28, and 42 days post-immunization (dpi) were used to detect GP5-specific IgG antibody by ELISA using purified GP5 protein as the antigen. At 14, 28, and 42 dpi, the antibody titer in the group inoculated with UEA-1/PLGA-SynORF5 was significantly higher than that of the group inoculated with pcDNA3.1-SynORF5 (*P* < 0.05) (Figure 3). The antibody titer in the group inoculated with UEA-1/PLGA-GP5 was also significantly higher than that immunized with GP5 (*P* < 0.05) (Figure 3). At 14, 28, and 42 dpi, no significant difference in GP5-specific antibody titers between groups immunized with UEA-1/PLGA-GP5 and PLGA-GP5 was observed. However, at 42 dpi, a significant difference in GP5-specific antibody titers was observed between groups immunized with UEA-1/PLGA-SynORF5 and PLGA-SynORF5 (*P* = 0.0026). Furthermore, at 42 dpi, UEA-1/PLGA-SynORF5 induced significantly higher GP5-specific antibody titers in mice compared with UEA-1/PLGA-GP5 (*P* = 0.0371). No detectable PRRSV-specific IgG antibodies (<1 : 80) were observed in sera from mice immunized with PBS during the experimental period.

**Figure 4:**
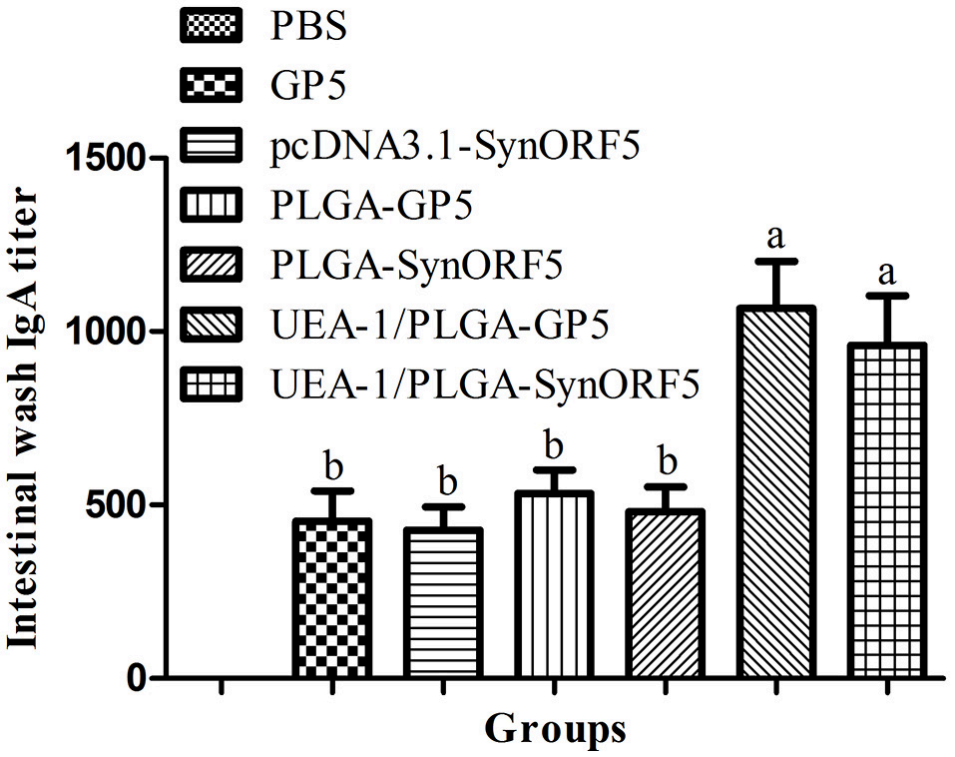
Mucosal immune response in mice induced by different PRRSV vaccine formulations. Female BALB/c mice (6–8 weeks old, *n* = 7 in each group) were orally immunized with 100 μL PBS, pcDNA3.1-SynORF5, GP5, PLGA-SynORF5, PLGA-GP5, UEA-1/PLGA-SynORF5, or UEA-1/PLGA-GP5 at 0 and 2 weeks. Intestinal samples were collected at 42 dpi to determine the GP5-specific IgA antibody by ELISA. Data represent the mean ± s.e.m. for 7 mice per group. Columns with different letters differ significantly (*P* < 0.05).

Intestinal lavage fluid samples obtained at 42 dpi were used to detect GP5-specific IgA antibody to evaluate the mucosal immune response induced by UEA-1 modified PLGA NPs. Mice immunized with UEA-1/PLGA-SynORF5 developed a significant higher IgA antibody titer (1: 960) than mice receiving pcDN3.1-SynORF5 (1: 426.67) or PLGA-SynORF5 (1:480) (*P* < 0.05) (Figure 4). Similar results of IgA antibody titer were observed among groups vaccinated with UEA-1/PLGA-GP5 (1: 1066.67), PLGA-GP5 (1: 533.33) or GP5 (1: 453.33) (*P* < 0.05). Furthermore, there was no significant difference in IgA antibody titer between groups inoculated with UEA-1/PLGA-SynORF5 and UEA-1/PLGA-GP5 (*P* = 0.5995). No detectable PRRSV-specific IgA antibodies (<1 : 20) were observed in sera from mice immunized with PBS. These results demonstrate that UEA-1 modified PLGA NPs could enhance mucosal IgA antibody titers induced by PRRSV DNA vaccine or GP5 subunit vaccine.

### Systemic and mucosal immune response in piglets

**Figure 5:**
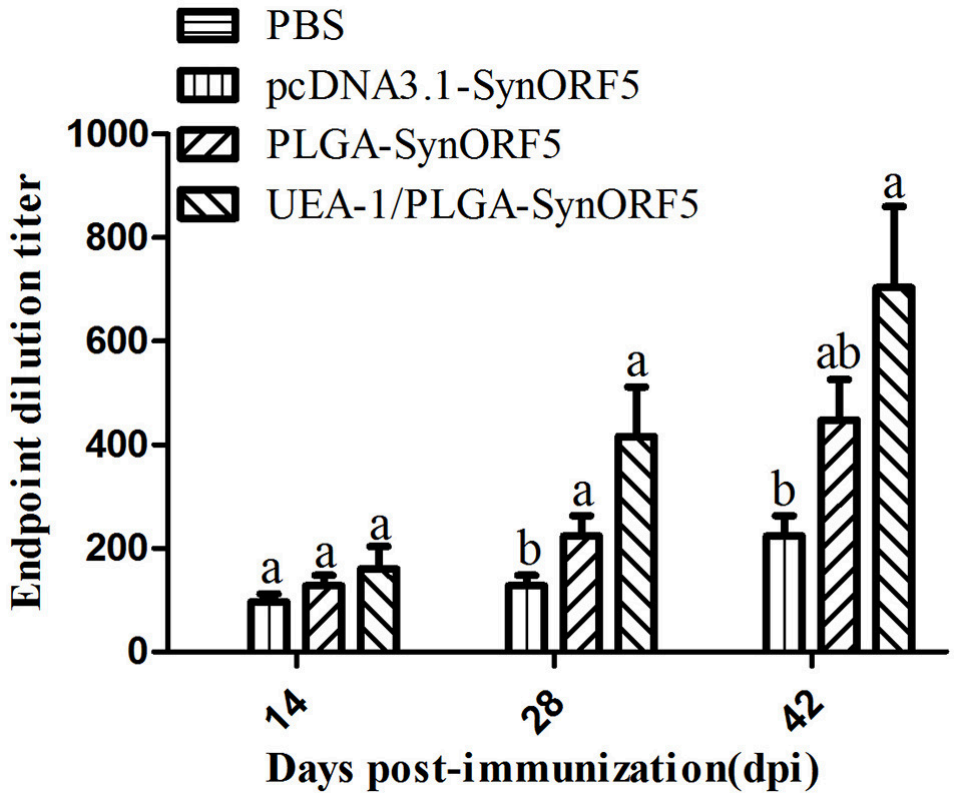
Systemic immune response in piglets induced by PBS, pcDNA3.1-SynORF5, PLGA-SynORF5 or UEA-1/PLGA-SynORF5. The immunization protocol is described in the Methods. Sera were collected at 14, 28 and 42 dpi to determine the GP5-specific ELISA antibody. Data are presented as the mean ± s.e.m. Columns with different letters differ significantly (*P* < 0.05).

Based on the results from mice immunization experiments, UEA-1/PLGA-SynORF5 was chosen to further evaluate the immunogenicity in piglets. Sera collected at 14, 28, and 42 dpi were used to detect GP5-specific antibody by ELISA using purified GP5 protein as the antigen. Anti-PRRSV GP5-specific antibodies in piglets vaccinated with pcDNA3.1-SynORF5, PLGA-SynORF5, or UEA-1/PLGA-SynORF5 were detected by ELISA at 14 dpi and increased following a booster inoculation (Figure 5). No detectable GP5-specific antibodies (<1 : 80) were observed in the PBS group at 14, 28, and 42 dpi. At 14 and 28 dpi, GP5 antibody titers in piglets immunized with UEA-1/PLGA-SynORF5 were significantly higher than that in piglets immunized with pcDNA3.1-SynORF5 constructs (*P* < 0.05). Although there were no significant differences between groups inoculated with PLGA-SynORF5 and UEA-1/PLGA-SynORF5 at 14, 28, and 42 dpi (*P* > 0.05), UEA-1/PLGA-SynORF5 induced numerically higher titers of GP5-specific IgG antibodies than PLGA-SynORF5.

**Figure 6:**
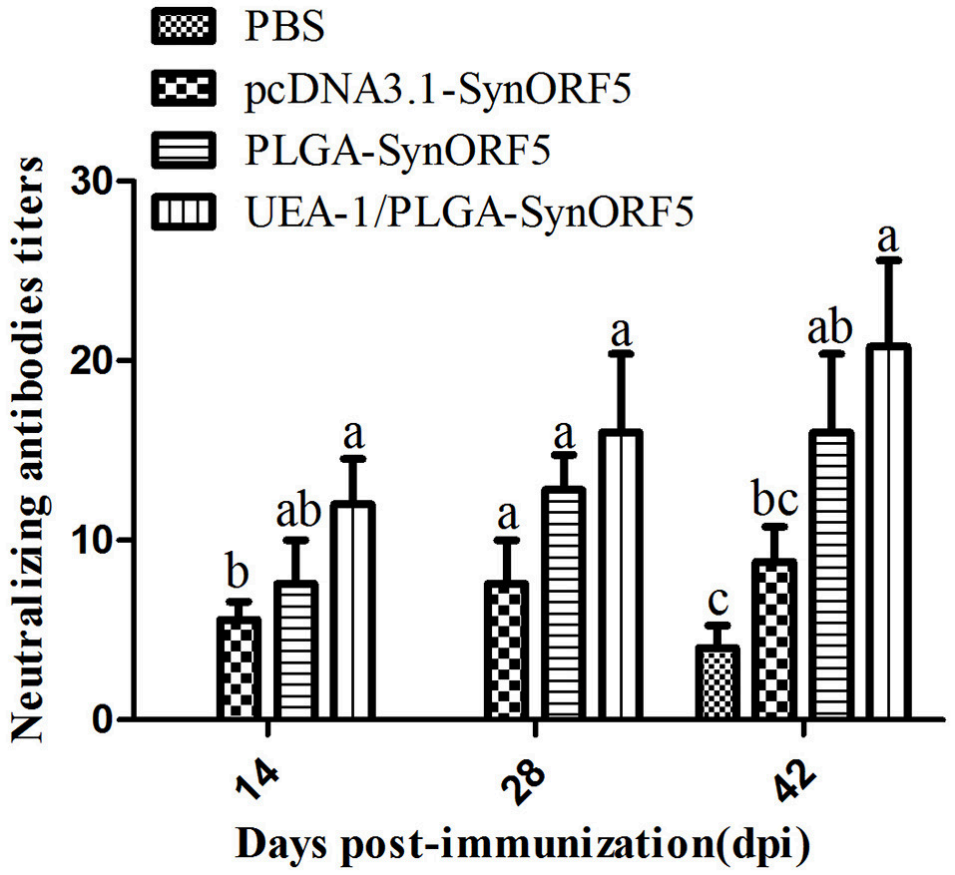
Neutralizing antibody titers in piglets induced by PBS, pcDNA3.1-SynORF5, PLGA-SynORF5 or UEA-1/PLGA-SynORF5. The immunization protocol is described in the Methods. Sera were collected at 14, 28, and 42 dpi to determine the neutralizing antibody titers. Data are presented as the mean ± s.e.m. Columns with different letters differ significantly (*P* < 0.05).

The neutralization capacity of sera from pigs was also investigated. PRRSV-specific neutralizing antibodies in pigs vaccinated with pcDNA3.1-SynORF5, PLGA-SynORF5 and UEA-1/PLGA-SynORF5 were detected at 14 dpi and elevated by 28 dpi (Figure 6). Meanwhile, no neutralizing antibodies (<1:2) against HP-PRRSV strain JSKM were detected in the PBS control group at 14 or 28 dpi. At 14 and 42 dpi, there were significant differences in the titer of neutralizing antibodies between the groups immunized with pcDNA3.1-SynORF5 and UEA-1/PLGA-SynORF5 (*P* < 0.05). At 14, 28, and 42 dpi, the neutralizing antibody titers detected in the group immunized with UEA-1/PLGA-SynORF5 were numerically higher than the group vaccinated with PLGA-SynORF5, but the differences were not statistically significant (*P* > 0.05).

**Figure 7:**
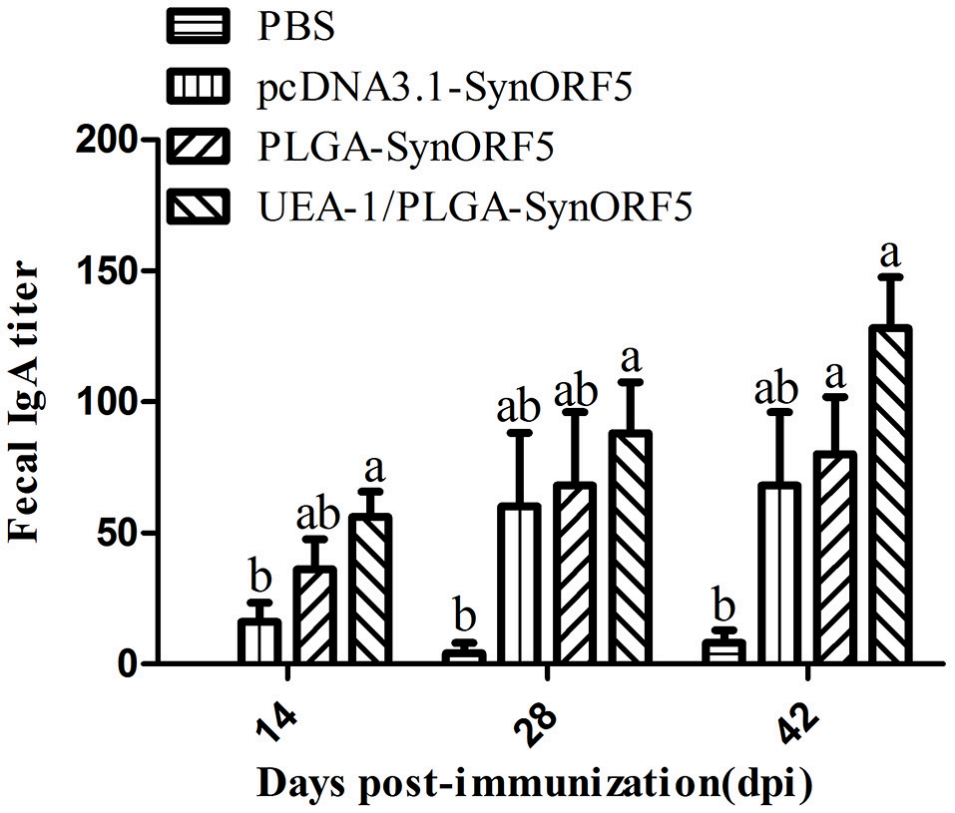
Detection of fecal GP5-specific IgA antibodies in piglets immunized by PBS, pcDNA3.1-SynORF5, PLGA-SynORF5, or UEA-1/PLGA-SynORF5. Data are shown as the mean ± s.e.m. Columns with different letters differ significantly (*P* < 0.05).

To analyze mucosal immune responses induced by UEA-1/PLGA-SynORF5, fecal samples were collected at 14, 28, and 42 dpi to detect the GP5-specific IgA antibodies. No detectable GP5-specific IgA antibodies (<1: 20) were observed in PBS groups at 14 dpi (Figure 7). At 14 dpi, UEA-1/PLGA-SynORF5 induced significantly higher GP5-specific IgA antibody titers than pcDNA3.1-SynORF5 constructs (*P* = 0.0118). Although there were no significant differences in GP5-specific IgA antibody titers between the groups immunized with PLGA-SynORF5 and UEA-1/PLGA-SynORF5 (*P* > 0.05) at each sampling time point, GP5-specific IgA antibody titers in groups vaccinated with UEA-1/PLGA-SynORF5 were numerically higher than groups receiving PLGA-SynORF5 at 14, 28, and 42 dpi.

**Figure 8:**
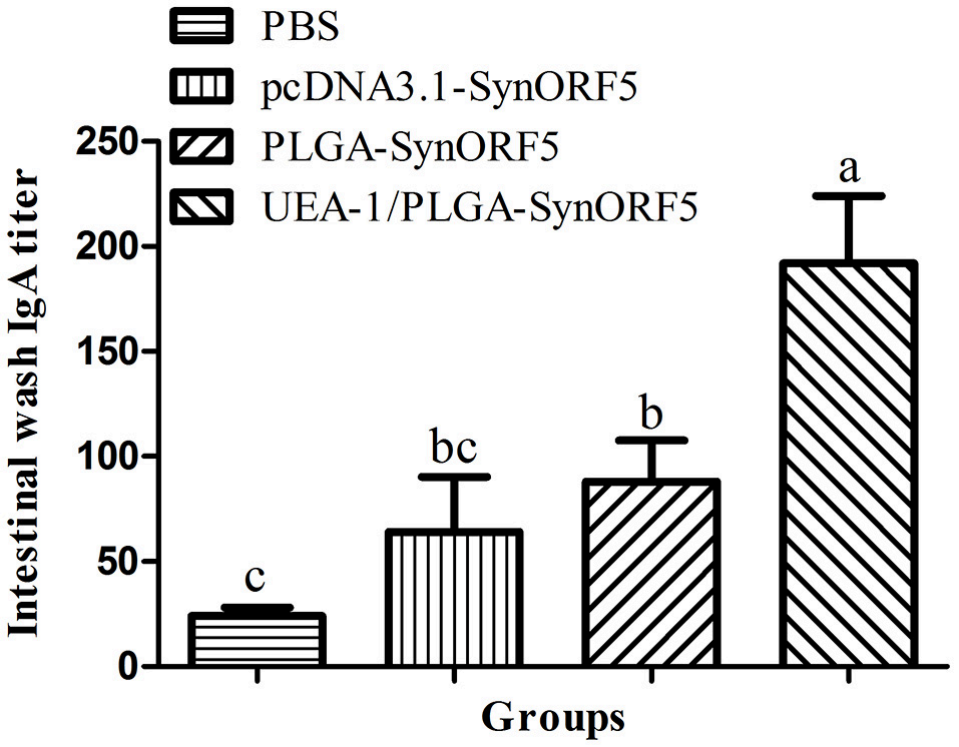
Detection of intestinal GP5-specific IgA antibody in piglets immunized by PBS, pcDNA3.1-SynORF5, PLGA-SynORF5 or UEA-1/PLGA-SynORF5. Data are shown as the mean ± s.e.m. Columns with different letters differ significantly (*P* < 0.05).

At 42 dpi all piglets were euthanized, and the intestines were sampled to determine GP5-specific IgA antibody titers. UEA-1/PLGA-SynORF5 induced the highest level of GP5-specific IgA antibody titers (1: 192) among all vaccinated groups (Figure 8). Additionally, there were significant differences in GP5-specific IgA antibody titers between groups immunized with UEA-1/PLGA-SynORF5 and any other construct (*P* < 0.05 for all).

## Discussion

It is becoming increasingly clear that mucosal immune response is the first line of defense against pathogens invading animals via mucosal sites (Neutra and Kozlowski, 2006). Mucosal vaccination can trigger mucosal immune responses, as well as systemic immunity, and induce mucosal IgA antibody and systemic IgG antibody (Holmgren and Czerkinsky, 2005).

Many studies have demonstrated the potential effectiveness of mucosal vaccines. It was reported that porous PLGA NPs coated with M cell homing peptide-coupled chitosan was a promising approach for the oral delivery of BmpB vaccine against swine dysentery (SD) with successful stimulation of specific IgG in serum and sIgA in feces and intestine (Jiang et al., 2014). Moreover, an M cell-targeting strategy facilitated mucosal immune response and enhanced protection against coxsackievirus B3 (CVB3) -induced viral myocarditis elicited by a chitosan-DNA vaccine (Ye et al., 2014).

Importantly, PLGA NPs could protect either the entrapped protein or the encapsulated DNA from the simulated harsh environment of the GI tract and released protein or DNA in a controlled manner (Adomako et al., 2012; Jiang et al., 2014; Ma et al., 2014). Here, we utilized PLGA NPs as the delivery system of a RRSV vaccine, which can protect PRRSV DNA or protein from low pH environment and enzymatic degradation as well as interact with the amine group of UEA-1 based on activation of its acid terminal group.

From the results of the entrapment efficiency of plasmid pcDNA3.1-SynORF5 and protein GP5, the EE of GP5 was as high as 97.47%, corresponding to the work completed by Ma et al. (2014) and Dwivedi et al. (2013). However, the EE of pcDNA3.1-SynORF5 was 52.24%. In consideration of a simple method to modify DNA vaccines, further efforts will be made based on the UEA-1/PLGA-SynORF5 construct to develop a more efficient PRRSV mucosal vaccine.

In this study, we first evaluated two different models of PRRSV vaccine, pcDNA3.1-SynORF5 or GP5 in mice. The constructs were encapsulated into PLGA NPs to prevent enzymatic degradation. UEA-1 was anchored onto PLGA NPs to confer M cell-targeting potential. As UEA-1/PLGA-SynORF5 induced higher level of systemic IgG and mucosal IgA antibody than UEA-1/PLGA-GP5, we chose UEA-1/PLGA-SynORF5 to evaluate the immune response following inoculation in piglets.

We demonstrated UEA-1 modified biodegradable PLGA NPs could improve systemic and mucosal immune responses induced by a PRRSV DNA vaccine or GP5 subunit vaccine in mice. Although there was no significant difference in PRRSV-specific IgA antibody titers between mice immunized with UEA-1/PLGA-SynORF5 and UEA-1/PLGA-GP5 NPs (*P* > 0.05), PRRSV-specific IgG antibody titers induced by UEA-1/PLGA-SynORF5 was significantly higher than that induced by UEA-1/PLGA-GP5 (*P* < 0.05). Furthermore, PLGA-SynORF5 or PLGA-GP5 could induce higher IgA antibody titers compared with pcDNA3.1-SynORF5 or GP5, respectively, however, the differences were not significant, which may be due to the properties of PLGA NPs. We speculated that PLGA NPs can protect antigens pcDNA3.1-SynORF5 or GP5 from enzymatic degradation and low pH gastrointestinal microenvironment (Lowe and Temple, 1994; Panyam and Labhasetwar, 2003; McNeil, 2005), but they could not efficiently transport antigen to M cells across the intestinal barrier. Furthermore, significantly higher IgA antibody titers were induced by UEA-1/PLGA-SynORF5 or UEA-1/PLGA-GP5 compared with PLGA-SynORF5 or PLGA-GP5, respectively. We speculated this finding was mainly due to the properties of UEA-1, which is capable of specific recognition and binding to M cells. *Ex vivo* ligated ileal loop assays confirmed our hypotheses.

Taken together, pcDNA3.1-SynORF5 induced relative high IgG levels in mice compared to PLGA-SynORF5, although the difference was not significant. However, contrasting results were observed in pigs. The difference in the immune responses between mice and piglets may be due to the differences of the GI microenvironment, additionally, in the mouse model, PLGA NPs may not have effectively protected pcDNA3.1-SynORF5 from low pH environments and enzymatic degradation. Results from experiments in piglets showed that UEA-1/PLGA-SynORF5 could induce elevated systemic and mucosal immune responses compared with pcDNA3.1-SynORF5 and PLGA-SynORF5. Although there were no significant differences in GP5-specific IgG antibody titers between groups immunized with UEA-1/PLGA-SynORF5 and PLGA-SynORF5 at each sampling point, mucosal IgA antibody titers induced by UEA-1/PLGA-SynORF5 were significantly higher than that of pcDNA3.1-SynORF5 and PLGA-SynORF5 (*P* < 0.05). This finding demonstrated that UEA-1 modified PLGA NPs could successfully enhance the mucosal immune response induced by pcDNA3.1-SynORF5. Therefore, UEA-1/PLGA NPs would be a promising delivery system for the development of a new PRRSV vaccine formulation.

Much effort has been made in past decades to develop effective approaches to induce mucosal immunity against PRRSV. A study on an adjuvanted PLGA NP-entrapped inactivated PRRSV vaccine (Nano-KAg) demonstrated that such a vaccine induces high levels of PRRSV specific IgA antibodies in the lungs of Nano-KAg and K-Ag vaccinated pigs through intranasal immunization. However, there was no difference in IgA antibody titers between groups intranasally immunized with Nano-KAg and K-Ag. In contrast, piglets inoculated with UEA-1/PLGA-SynORF5 induced significantly higher PRRSV IgA antibody titers in the intestinal tract compared with pigs vaccinated PLGA-SynORF5 and pcDNA3.1-SynORF5. Unfortunately, we did not detect IgA antibody titers in the lungs. The ORF5-encoded major envelope glycoprotein (GP5) is one of the key immunogenic proteins of PRRSV and is the leading target for the development of the genetic engineering vaccines against PRRS, so we chose GP5 in the research. In further studies, we intend to develop a more efficient PRRSV mucosal DNA vaccine by inserting other genes encoding immunogenic proteins or heterologous PRRSV ORF5 genes. Furthermore, we will simultaneously evaluate PRRSV-specific IgA antibodies in the lungs, as well as cellular immune responses, to comprehensively analyze the potential of the PRRSV DNA vaccine to induce systemic and mucosal immune responses.

Based on our present study, we believed that this M cell-targeting strategy may be applied as a universal platform for mucosal DNA vaccine development.

## Author contributions

LD, BL, and KH designed the experiment. Sampling of porcine serum, feces and intestines were mainly performed by LD, FP, AM, ZY, and WY. LD and XX analyzed the results with guidance from BL, and KH and wrote the main manuscript text. All authors took part in discussion and interpretation of results. All authors read, advised and approved the final manuscript.

### Conflict of interest statement

The authors declare that the research was conducted in the absence of any commercial or financial relationships that could be construed as a potential conflict of interest.
